# Losses of Life Expectancy and Productivity Associated with COVID-19 Pandemic in Canada: Policy Implication for Future Communicable Disease Control

**DOI:** 10.3390/ijerph20032419

**Published:** 2023-01-29

**Authors:** Fuhmei Wang, Jinwei Lui, Jung-Der Wang

**Affiliations:** 1 Department of Economics, College of Social Science, National Cheng Kung University, Tainan 704, Taiwan; 2 Department of Public Health, College of Medicine, National Cheng Kung University, Tainan 704, Taiwan; 3 Department of Occupational and Environmental Medicine, National Cheng Kung University Hospital, College of Medicine, National Cheng Kung University, Tainan 704, Taiwan

**Keywords:** difference-in-differences, life expectancy, Canada

## Abstract

This research examines whether the Coronavirus disease 2019 (COVID-19) did harm to the population’s health through comparing the changes in the life expectancy of Canadians with those of Australians over the period from March 2019 to February 2021 by using a difference-in-differences (DID) estimation method. We found that the pandemic did cause differences in life expectancies between Canada and Australia, probably because of different initial control policies for COVID-19. This study uses the indicator of disability-adjusted life years (DALYs) to measure the societal health burden, which was corroborated by estimating temporal productivity loss (TPL) and permanent productivity loss (PPL) based on the human capital approach (HCA) using data from Health Canada. The societal health burden in Canada amounted to 6.493 DALYs per 1000 male persons and 5.316 DALYs per 1000 female persons. The economy’s permanent productivity loss was around USD 5.3 billion, while the temporary productivity loss was around USD 3 billion from February 2020 to April 2022. The sum of the above two losses amounted to 0.477% of the GDP in 2019. Swift and decisive decisions at the very early stage of a pandemic can nip contagions in the bud before numbers get out of hand and would be less damaging to people’s health and the economy, as seen in Australia, in contrast to what happened in Canada. We thus recommend that such policies plus telecommunication systems in healthcare services be implemented early on to cope with the future outbreak of any emerging infectious diseases such as COVID-19.

## 1. Introduction

Coronavirus disease of 2019 (COVID-19) was reported for the first time in Wuhan, China, near the end of December 2019. The virus spread worldwide rapidly (https://www.who.int/europe/emergencies/situations/covid-19, accessed on 1 December 2022). The pandemic outbreak of COVID-19 has resulted in enormous numbers of mortality and morbidity cases across different ages, sex, ethnic origins, and countries [1,2]. The first case of COVID-19 was reported on 25 January 2020 in Australia, while the first case in Canada was reported on 26th January 2020. Before the availability of vaccines in July of 2021, Canada reported a total of 1,419,964 incidence cases and 26,016 mortality cases, while the numbers in Australia, in contrast, were 30,562 and 910 cases, respectively [3,4].

### 1.1. Literature Review

Studies have investigated the medical transmission routes and system developments of COVID-19 [5,6,7]. Hospitalized and/or infected patients with COVID-19 could suffer from loss of productivity in the labor force temporarily, due to absenteeism from work, or permanently, due to premature death. The harmful impacts of the pandemic on the productivity of various countries have been reported and evaluated in terms of gross domestic product (GDP) [8,9,10]. The impacts on human capital stratified by age in the US and Italy have been estimated [11,12]. However, while mortality on gender difference was observed [13], there seems to be a knowledge gap with regard to different productivity losses because of different sexes [11]. The impacts of gender on the outbreak and the comprehensive exploration on health and economic burdens from a societal perspective, stemming from quarantine, hospitalization, and death, have attracted our attention.

### 1.2. Selection of Investigated Countries

Canada and Australia are both former British colonial regions and currently are federal states with constitutional monarchies with a very similar cultural background, universal healthcare systems, dates of availability of vaccines and therapeutic medications for COVID-19, as well as similar economic performances in terms of GDP (gross domestic product), well-developed international trade networks, and so on. Prior to the COVID-19 pandemic, the life expectancies of Australia and Canada were around 82.9 and 82.05, respectively, in 2019. Although Canada’s population of 35.2 million is larger than Australia’s 23.1 million, both are spread across massive uninhabitable continents with low population densities at four persons per square kilometre in Canada and three persons per square kilometre in Australia. Some 64% of Australians live in the five largest cities, while 45% of Canadians live in the six largest metropolitan areas. The top 10 causes of death were also similar, beginning with malignant neoplasms, ischemic heart disease, cerebrovascular disease, pneumonia, and dementia, etc. [14,15].

The Australian government immediately closed their borders and imposed external and internal travel restrictions and a quarantine for 14 days for travelers, proceeded with a cautious school closure and movement to online schools, and required the wearing of masks in the workplace. In contrast, the Canadian government postponed such stringent policies until March of 2020. As time went by, the cumulative numbers of incidence and mortality cases in Australia were much lower than those in Canada before vaccines were introduced into both countries in July 2021, which in turn reflects a difference in infection rates.

Based on the experiences of Canada and Australia, this work endeavors to corroborate whether COVID-19 did, in fact, affect the population health by using the method of difference-in-differences (DID). The DID method has been widely applied to assess the changes caused by an event between the study and control groups [16]. To evaluate the possible changes in health status, represented by life expectancy [17], this research defines the pre-event period, 2019, as the year before the pandemic and the post-event period as the year 2020 and afterwards.

### 1.3. The Objectives

Using the indicator of disability-adjusted life years (DALYs), this research further calculates the productivity loss estimated by forgone earnings during the COVID-19 pandemic from a societal perspective, which would be a lower bound of saving for preventing large scale infectious diseases in the future.

Our study contributes to and improves on earlier work in several ways. First, we examined the differences in population health between the two countries, Canada versus Australia, over the period of COVID-19 from a societal perspective [18]. Second, this study is among the first to corroborate the burden of DALYs with foregone earnings to report the impact on human capital across different age groups [11]. In particular, this research provided estimations for verifying the sex differences of the outbreak. Third, we calculated the temporary and permanent productivity losses for people aged 15 years and above based on the 2021 Canada weekly wage across different age and sex groups.

In the following, this article describes the calculation process, analyzes how the COVID-19 pandemic affected the population’s health and the economies, and provides policy implications.

## 2. Materials and Methods

Our hypothesis was that different initial control policies for COVID-19 would impact population health and societal productivity differently. Based on similar backgrounds of demographic, cultural, and universal coverage healthcare systems, we applied a DID model to examine how life expectancies were affected in Canada versus Australia first. As Australia is less affected, we went on estimations of burden of disease via the DALY (disability-adjusted life year) method and actual productivity loss based on a human capital approach in Canada; namely, we hope to quantify the magnitude of harm or damage by COVID-19 from a societal perspective to propose relevant policies for the improvement of the future control policy of the infectious disease epidemic.

### 2.1. The Difference-in-Differences Specification

Prior to the COVID-19 pandemic, Australia and Canada had similar life expectancies (LEs) of 82.9 and 82.05 years, respectively, in 2019, which were collected and considered as a baseline for each country. The LEs of the observed period from March 2020 to February 2021 of the two compared countries were then collected to examine whether different initial control policies on COVID-19 in Canada versus Australia affected the population’s LEs differently. We presume that there was neither infection nor mortality in both countries due to COVID-19 before February of 2020. This research examines whether COVID-19 did harm the population’s health by comparing the changes in the outcome variable, and life expectancy of the study country, Canada, with those of the control country, Australia. We collected the monthly life expectancy of Canadians and Australians from March 2019 to February 2021 according to Statistics Canada and the Australian Bureau of Statistics [14,15]. To guarantee the accuracy of the DID estimate, the composition of individuals of the two groups is assumed to remain unchanged before COVID-19, over the observed period from March 2019 to February 2020.

The specification of the DID model was set as follows:

(1) $$\;L_{ijt}=\beta D_{it}+\gamma S_{i}+\phi t+\alpha +\varepsilon_{it}$$

where *L_ijt_* denotes the life expectancy of an individual *j* at different ages *i* of the study and control countries at time *t*; *D* denotes the presence of COVID-19 (0 stands for months without COVID-19 before February 2020, namely, the months from March, 2019 to February 2020; 1 stands for months with COVID-19 after February 2020, namely, the months from March 2020 to February 2021); *S_i_* denotes the dummy variable for sex (1 stands for female; 0 stands for male) at different ages *i* of the study and control countries; *α* represents time-invariant individual heterogeneity, and *ε_it_* are the idiosyncratic errors. We applied ordinary least squares to the above formula using Stata 17. The coefficient *β* represents the differences in life expectancy between Canadians and Australians due to the occurrence of COVID-19, whereas the coefficient *γ* represents the differences in life expectancy between females and males. The coefficient *ϕ* represents the differences in life expectancy between the pre-COVID and post-COVID periods for the study country, Canada. Intercept *α* depicts the life expectancy of an individual *j* male in Australia at different ages. Table 1 presents the summary monthly statistics of Canada and Australia from March 2019 to February 2021, stratified by age and sex. Variables Sex, *S*, and time, *t*, in Equation (1) with median, mean, Std. Dev. min, max at the values of 0.5, 0.5, 0.5, 0,1, respectively.

### 2.2. Estimation of Societal Health Burden in Terms of DALYs

DALYs is a time-based measure composed of years of life lost (YLLs) due to premature mortality and years living with disability (YLDs) for incident cases [19]. This method is appropriate for quantifying the burden of disease and the productivity loss from a societal perspective for a large-scale infectious disease. Nurchis et al. has applied DALYs to calculate the societal burden of COVID-19 in Italy, one of the most affected countries [11]. The YLLs due to COVID-19 were specified as the age at which death occurred and were calculated as the number of deaths multiplied by a loss function identifying the years lost for health. The loss function was based on the frontier national life expectancy projected for 2019, the year before the pandemic. The disability weight is defined according to the Global Burden of Disease (GBD) project for different diseases as categorized by the World Health Organization (WHO) [20,21,22]. COVID-19 generally results in mortality through pneumonia, of which GBD sets the disability weight at 0.133. Prevalent YLDs were calculated as the prevalence of each infected person multiplied by its disability weight 0.133 [23].

### 2.3. Estimation of Temporary and Permanent Productivity Losses

We collected mortality and infection monthly data over the period from 1 February 2020 to 30 April 2022 and calculated the temporary and permanent productivity losses due to COVID-19 in Canada. Based on the data reported by Statistics Canada [3], we calculated the permanent lifetime productivity loss for premature mortality cases in different age bands for men and women. The calculation process was as follows: First, we set the difference between the statutory retirement age, 69, and the age of death as the working years lost. Second, we calculated the average annual income in different age bands for men and women by adjusting the labor participation rates and employment rates. Third, the lifetime productivity loss was determined as the sum of the present value of annual adjusted income by using a discounting rate of 0.03. For calculating the lifetime productivity of the labor force, the 0.03 discounting rate fitted the real rate of return used to discount future cash flows back to their present value [24]. The economy’s productivity loss was the sum of the estimated cases of death multiplied by the loss in lifetime earnings across different sex and age bands.

On the other hand, the population infected by COVID-19 and unable to work would temporarily lose their earnings. Following Nurchis et al., the period of absenteeism from work was set as two weeks [11]. Collecting the average two-week wages for the different age bands, and multiplying them by the estimated number of incidence cases and summing up the loss in earnings yielded the total temporary productivity loss.

## 3. Results

### 3.1. COVID-19 Affects Life Expectancy Significantly

Table 2 presents the estimation results of the DID model on life expectancy. Coefficient *β* represents that the life expectancy in Canada was significantly less than that in Australia due to the occurrence of COVID-19 by 0.966, 0.88, 0.781, 0.723, 0.655, 0.374, and 0.049 for the age groups 0–19, 20–29, 30–39, 50–59, 60–69, 70–79, 80+, respectively. Though the pandemic-related mortality appeared in older ages in most countries, the overall reduction in life expectancy seemed higher in younger ages. Coefficient *γ* indicates that the life expectancies of women across different age groups were greater than those of males by 1.091 to 3.624 years in both countries, which seemed to provide support to the validity of this model. Coefficient *ϕ* represents that the COVID-19 pandemic significantly reduced the expected life expectancies across the different age groups in Canada by 0.181 to 0.209 years. The intercept *α* represents men’s expected life expectancies at different age groups in the control country, Australia. Through the DID model construction, we found that the pandemic did cause differences in life expectancies between the study and control countries.

### 3.2. The Pandemic Resulted in Societal Health Burden

Through the construction of the DID model, we found that the pandemic did cause differences in life expectancies between Canada and Australia probably because of different initial control policies for COVID-19. We then further calculated the societal health burden due to the pandemic through infection and mortality data over the observed period with COVID-19 from February 2020 to April 2022 in Canada. Table 3 presents the influences of COVID-19 on YLLs, YLDs and DALYs across different sex and age groups. The health burden of YLLs and DALYs increased with age. Males had higher DALYs than females under the age of 70. However, over the age of 80, women had higher mortality, vulnerability, and DALYs than men in Canada, which was in contrast to early assumptions [13]. This pandemic caused a health burden of 6.493 DALYs per 1000 men with 5.897 YLLs plus 0.596 YLDs, while those of women were 5.316 DALYs per 1000 with 4.654 YLLs and 0.662 YLDs. Higher losses in men than women probably resulted from gender differences [11,25]. We also found that in Canada, the older the age, the higher the proportion of YLL in the total number of QALY for both men and women. Above age 70, YLLs occupied more than 90% of DALYs in each age band due to the high mortality rate of the elderly infected with COVID-19. These findings are similar to those in Italy, one of the most affected countries [11].

### 3.3. Substantial Temporary and Permanent Productivity Losses

An infected employee could suffer from temporary productivity loss due to absenteeism from work and permanent productivity loss due to premature death. The length of temporary absenteeism from work was set as two weeks because employees might need to be quarantined and/or might experience COVID-19 symptoms and seek medical treatment for recovery. Table 4 presents the estimated number of cases and temporary productivity loss per infected employee, which were summed up to yield the total temporary productivity loss and the percentages of total temporary productivity loss relative to the GDP across different sex and age bands. The estimated temporary productivity loss increased with age, reached a peak of around 0.042 (0.039) percent of the GDP in 2019 for 35–44 years old male (female) workers, and then decreased with age until 69 years old, down to around 0.007 (0.005) percent of the GDP over the period from 1 February 2020 to 30 April 2022.

When an infected employee dies, his/her productivity is lost permanently. Table 3 presents the estimated permanent productivity loss for different sex and age bands. Male workers in the fifth age band (i.e., 55–64) had the highest permanent productivity loss, which amounted to 0.0503 percent of the GDP in 2019 due to the higher number of estimated cases compared to the other age bands. In contrast, female workers in the fourth age band (i.e., 45–54) had the highest permanent productivity loss, which amounted to 0.0179 percent of the GDP in 2019 due to their higher average wages compared to the other age bands.

The total permanent productivity loss was approximately USD 5.3 billion, while the total temporary productivity loss was around USD 3 billion. The sum of temporary and permanent productivity losses amounted to about USD 8.3 billion and approximately 0.477% of the GDP in 2019.

## 4. Discussion and Policy Implications

Although we all know that a pandemic infectious disease would affect a population’s health and result in a shortening of life expectancy (LE), our aim is to quantify the magnitude of effects because of different initial control policies in different countries for a pandemic infectious disease. Using COVID-19 as a real-life example, we chose Canada and Australia because of their similarities in backgrounds of demographic, cultural, and universal coverage of the healthcare system, which would affect the life expectancy the most. Prior to the COVID-19 epidemic, the life expectancies of Australia and Canada were 82.9 and 82.05 years, respectively, in 2019. We found that the reduction in life expectancies associated with COVID-19 seemed heavy in all age groups (Table 2), which corroborated those reported by Schöley et al. [26]. The constructed difference-in-differences (DID) model successfully quantified the magnitude of loss of life expectancy because of different initial control policies between Canada and Australia. Canada did show a higher loss of life expectancy than that of Australia, including those below age 70 (Table 2), which lead us to quantify the productivity loss from a societal perspective. The health losses reflected in terms of DALYs increased with age and hurt the elderly population the most and were in line with the characteristics of this pandemic (Table 3). As an associated consequence, the impact of COVID-19 on the labor force resulted in employees being quarantined, hospitalized, or deceased, leading to losses in temporary or permanent productivity. Table 4 shows that the population aged 15–69 represented the majority of the labor force and were vital to sustainable contributions to productivities and economic growth. In particular, male and female employees in the third age band (i.e., 35–44) had the highest loss in temporary earnings compared to those in other age bands. In contrast, male employees aged 55–64 suffered from the highest loss in lifetime productivity due to premature mortality.

The outbreak curves of the two countries looked very similar to what the CDC of the EU warned from the very beginning [27]. Namely, swift and decisive decisions at the very early stage of a pandemic can nip contagions in the bud before the numbers get out of hand, and would be less damaging to people’s health and the economy as seen in Australia in contrast to what happened in Canada. We thus recommend that such policies be implemented early on for healthcare services to cope with the future outbreak of any emerging infectious diseases such as COVID-19.

Nurchis et al., calculated the productivity loss by age group and cast doubt that there are gender differences in mortality and vulnerability to the disease [11]. This research constructed the DID model to estimate the differences in sex and confirmed that women had a longer life expectancy than men in both Canada and Australia. Moreover, men appeared to suffer from a higher YLL and higher temporary and permanent productivity losses than women in Canada during this outbreak.

As the wage data collected from Statistics Canada is generally based on the weekly wage, which is the base pay and does not include bonuses, compensation, etc., it might have underestimated the losses in productivity. Moreover, our estimations using the human capital approach would generally be of a lower bound compared to the willingness-to-pay approach that is the foundation of welfare economics [28].

## 5. Conclusions

As the early establishing of emergency quarantine action is very crucial, each country must communicate with their neighboring countries to set up such a policy, which would require close international cooperation under the International Health Regulations. In addition to establishing quarantine policies as early as possible, there are other measures that may ameliorate the loss of productivity under the outbreak of communicable diseases, such as the possibility of working at a remote office or from home, to mitigating the risk of COVID-19 onward transmissions and associated morbidity and mortality [27]. Providing adequate personal protective equipment, safe working conditions, and improving the general health of employees could possibly reduce losses in productivity and health and economic burdens [27,29,30].

## Figures and Tables

**Table 1:** Summary Monthly Statistics of Canada and Australia from March 2019 to February 2021, stratified by age and sex.

	Life Expectancy	Median	Mean	Std. Dev.	Min	Max
Ages						
0–19		82.3	82.5	1.9	76.6	84.8
male		80.8	80.7	0.5	79.6	81.3
female		84.4	84.3	0.5	83.3	84.8
20–29		62.8	63	1.8	60.2	65.2
male		61.3	61.3	0.5	60.2	61.7
female		64.9	64.8	0.5	63.8	65.2
30–39		53	53.3	1.7	50.7	55.4
male		51.7	51.7	0.4	50.7	52.1
female		55	55	0.4	54	55.4
40–49		43.4	43.7	1.6	41.2	45.6
male		42.2	42.1	0.4	41.2	42.5
female		45.3	45.2	0.4	44.3	45.6
50–59		34	34.3	1.5	31.9	36
male		32.9	32.8	0.4	31.9	33.2
female		35.7	35.7	0.4	34.7	36
60–69		25	25.3	1.3	23.1	26.8
male		24.1	24	0.4	23.1	24.4
female		26.6	26.5	0.4	25.6	26.8
70–79		16.7	16.9	0.9	15.2	18
male		16	16	0.3	15.2	16.2
female		17.9	17.9	0.2	17.2	18
80+		9.4	9.6	0.57	8.6	10.4
male		9.2	9.1	0.2	8.6	9.2
female		10.2	10.2	0.2	9.6	10.4

**Table 2:** Regression coefficients (with standard errors of means in parentheses) estimated from model constructions of difference-in-differences comparing the life expectancies of Canadians and Australians.

	Ages	0–19	20–29	30–39	40–49	50–59	60–69	70–79	80+
Coefficients									
*β*		−0.996 *	−0.88 *	−0.781 *	−0.723 *	−0.732 *	−0.655 *	−0.374 *	−0.049
		(0.029)	(0.029)	(0.029)	(0.029)	(0.028)	(0.028)	(0.027)	(0.026)
*γ*		3.624 *	3.524 *	3.295 *	3.067 *	2.832 *	2.503 *	1.899 *	1.091 *
		(0.029)	(0.029)	(0.029)	(0.029)	(0.028)	(0.028)	(0.027)	(0.026)
*ϕ*		−0.209 *	−0.209 *	−0.209 *	−0.207 *	−0.204 *	−0.199 *	−0.191 *	−0.181 *
		(0.029)	(0.029)	(0.029)	(0.029)	(0.028)	(0.028)	(0.027)	(0.026)
*α*		81.32 *	61.71 *	52.16 *	42.61 *	33.3 *	24.43 *	16.25 *	9.213 *
		(0.029)	(0.029)	(0.029)	(0.029)	(0.028)	(0.028)	(0.027)	(0.026)

* *p*-value is significant at <0.001; R-squared: 0.98; observed period: March 2019 to February 2021; the number of observations was 96. Meanings of coefficients: *β*: the differences in life expectancy between Australians and Canadians that were attributable to the occurrence of COVID-19. *γ*: the differences in life expectancy between females and males. *ϕ:* the differences in life expectancy of Canadians before and after the occurrence of COVID-19. *α*: the life expectancy of males in Australia.

**Table 3:** The influences of COVID-19 on years of life lost (YLLs), years living with disability (YLDs), and disability-adjusted life years (DALYs) in Canada.

	Loss in DALYs	Male (per 1000)			Female (per 1000)		
Ages		YLLs	YLDs	DALYs	YLLs	YLDs	DALYs
0–19		0.032	0.134	0.166	0.041	0.129	0.17
20–29		0.117	0.131	0.248	0.077	0.154	0.231
30–39		0.24	0.113	0.353	0.157	0.139	0.296
40–49		0.411	0.086	0.497	0.248	0.104	0.352
50–59		0.829	0.064	0.893	0.538	0.068	0.606
60–69		1.33	0.036	1.366	0.797	0.033	0.83
70–79		1.454	0.018	1.472	1.003	0.016	1.019
80+		1.484	0.014	1.498	1.793	0.019	1.812
total		5.897	0.596	6.493	4.654	0.662	5.316

**Table 4:** Estimated temporary and permanent productivity losses (TPL and PPL) at different ages in Canada from February 2020 to Apr. 2022 based on the 2019 GDP (gross domestic product).

Sex	Ages	TPL (Temporary Productivity Loss)			Total TPL/GDP (%)	PPL (Permanent Productivity Loss)			Total PPL/GDP (%)
		Estimated No. Incidence Cases	TPL per Case	Total TPL (× 10^3^ US$)		Estimated No. Mortality Cases	PPL per Case	Total PPL (× 10^3^ US$)	
**Male**	15–24	254,253	685	174,066	0.0100	38	247,055	9388	0.0005
	25–34	314,358	2000	628,716	0.0361	123	1,029,123	126,582	0.0073
	35–44	272,658	2692	734,079	0.0422	279	1,251,061	349,046	0.0201
	45–54	229,905	3104	713,590	0.0410	713	1,091,788	778,445	0.0447
	55–64	191,017	2585	493,706	0.0284	2035	430,490	876,047	0.0503
	65–69	63,522	1862	118,249	0.0068	1205	82,011	98,823	0.0003
**Female**	15–24	277,983	600	166,790	0.0096	27	221,319	5976	0.0057
	25–34	379,448	1615	612,955	0.0352	80	779,999	62,400	0.0036
	35–44	341,384	2000	682,768	0.0392	173	834,830	144,426	0.0083
	45–54	273,217	2162	590,569	0.0339	445	698,061	310,637	0.0179
	55–64	202,263	1665	336,846	0.0194	1192	231,758	276,255	0.0159
	65–69	63,142	1362	85,970	0.0049	702	38,120	26,760	0.0015

## Data Availability

The data underlying the results presented in the study are from third party web resources and are available from the URL: https://www150.statcan.gc.ca/t1/tbl1/en/tv.action?pid=1410001701. The statistics are open for the public. (accessed on 14 December 2022).
